# Adolescent pregnancy in sub-Saharan Africa – a cause for concern

**DOI:** 10.3389/frph.2022.984303

**Published:** 2022-12-02

**Authors:** Niren Ray Maharaj

**Affiliations:** Department of Obstetrics and Gynaecology, University of KwaZulu-Natal, Durban, South Africa

**Keywords:** adolescents, sexually transmitted infections, HIV, contraception, pregnancy

## Introduction

It is estimated that adolescent girls in low- and middle-income countries (LMICs) have approximately 21 million pregnancies each year (1). Adolescent pregnancy, which occurs amongst adolescent girls aged between 10 and 19 years, is associated with various public health concerns and increased risks of maternal mortality, low birthweight, and other severe neonatal complications (2). They also have an unfavourable impact on the mental, physical, and social wellbeing of adolescents, and remain a leading cause of death among adolescent girls globally (2).

Although a decline in adolescent birth rates (ABR) has been observed globally, sub-Saharan Africa (SSA) continues to have twice the global average, with over 100 births per 1,000 women, in 2021 (3). The estimated actual number of births among 15–19-year-olds was 6 114 000 and 332 000 among younger adolescents aged 10–14 years in SSA in 2021 (3). Africa has a higher proportion of youth compared to any other continent; and adolescent pregnancy rates are therefore likely to increase further in countries in SSA (4). Adolescent pregnancies are therefore a significant health concern and have been recognised as a key objective in the United Nations Sustainable Development Goals (SDGs) (5). The contributing factors for high ABR in SSA are heterogenous and differ amongst countries in SSA. Existing data on the contributing factors for adolescent pregnancies in SSA emanate from country specific studies and collective data is limited. However, a few studies that combined data from single country studies on the determinants of adolescent pregnancy have been conducted (6–8). Collectively, these studies suggest that inter alia, socio economic status, lack of parental communication and support, early marriage, religion, and low educational status of adolescents are contributing factors.

### Economic, sociocultural, and environmental factors

Poverty is generally considered as an indicator of the economic status of a country. Studies from Ghana (9), South Africa (10) and Tanzania (11) demonstrate the relationship between poverty and coerced sexual relations with older men, as a means for adolescents to meet their basic financial needs. Adolescent girls may also become pregnant intentionally to receive government support grants intended for teenage mothers, without considering the consequences of their actions (12). Nigeria, the country with the largest economy in Africa had an adolescent pregnancy rate of 106 adolescent births per 1,000 population in 2021 and shows an increasing trend (13). In South Africa (SA), which has the second largest economy in Africa, the ABR is also high (14). In SA, the lack of adequate reproductive health services and appropriate sexuality education are part of the composite etiology that contributes to increasing ABR's (15). Between 2017 and 2021, births among girls aged 10–14 years and 15–19 years increased in SA by 48.7% and 17.9% respectively, and are likely to increase (16). In contrast, in Burundi, which has the lowest gross domestic product per capita in Africa (17), the adolescent fertility rate (births per 1,000 women ages 15–19) was 58/1,000 compared with the total fertility rate of 5.5/1,000 women in that country (18). These comparisons suggest that the economic status of countries in Africa remains part of a composite etiology, which may also include power imbalances, gender-based violence, substance abuse, lack of access to termination of pregnancy services, negative attitudes of caregivers and inadequate reproductive health education.

It has also been suggested that lower levels of education may be associated with adolescent pregnancies. In Niger, Mali, and Chad, where the adolescent fertility rates are amongst the highest in the world, the expected years of schooling attained by girls between the ages of 4 and 17 is fewer than seven years (2). On the contrary, other studies suggest that higher levels of education are likely to be associated with a lower likelihood of having a first adolescent pregnancy, particularly in SSA (4, 7). Adolescents with higher levels of education are more likely to delay the onset of sexual relations and marriage; and are more informed about their rights, reproductive health, timing of marriage and pregnancy (19).

Child marriages are also implicated as a contributing cause for high ABR's in SSA, particularly in the Congo and Central Africa (20). The Congo has one of highest rates of child marriage globally, with one in three girls married before age 18, and 7% married before the age of 15 (20). Similarly, other studies have also shown an association between child marriage and adolescent pregnancy in African countries (21, 22). Most girls who experience child marriage have low levels of education, live in poor households and often in rural areas, increasing their odds of engaging in behaviours that put them at risk of pregnancy (23). Data also suggests the association of child marriages with first pregnancy among adolescent girls in SSA (6). Pregnancies in these marriages may occur because of pressure from partners or family members to start families earlier or to prove reproductive potential. In most sub-Saharan African countries, adolescent girls may face social pressure to marry and, once married, to have children (4). In parts of South Africa, cultural practices like “ukuthwala” also lead to adolescent pregnancies. This practice involves the arranged marriage of girls below the age 18 mostly to older men, without the bride's consent (24). This type of marriage is in violation of the country's national law, as well as regional and international instruments to which this country is a party (24).

### Individual factors

Individual perceptions about abstinence from sexual intercourse, early sexual debut, and negative perceptions about contraceptive usage among adolescents may contribute to adolescent pregnancies. Sexual coercion, low or incorrect use of contraceptives, and low self-esteem have also been suggested as contributing factors (6–8). Other personal factors may include stigma, fear of negative attitudes from parents and elders in the community and discrimination by healthcare providers. It has been suggested that healthcare providers may not appreciate the fears of adolescents regarding contraception or reproductive health issues, and adolescents themselves may not appreciate comprehensive sexuality education (10). Adolescents may also perceive contraceptive usage as a reserve for married couples, thereby contributing to low contraceptive uptake and resultant pregnancies (10). Additionally, a lack of awareness, misconceptions and poor knowledge about the range and use of contraceptive methods exists amongst some adolescents (25). Alternatively, some adolescents may desire pregnancy despite a suitable knowledge and available access to contraception. Career plans may also be affected, and poorly educated, unemployed and grant dependent youngsters pose an economic burden on the fiscus in the long term.

### Health related factors

Teenagers may also face challenges in accessing reproductive health care in their communities. In some rural areas, the sparsity of clinics, long distances, and lack of transport may present barriers to access to reproductive health services. Judgemental attitudes of staff, particularly to teenagers seeking contraception or termination of pregnancy services should be avoided. Poor staff attitudes at termination of pregnancy clinics, may drive teenagers to seek “back street” terminations, with potential morbidity and even mortality.

The Covid epidemic may have exacerbated the problem further, with resultant school closures and access to contraception and healthcare services being unavailable or restricted during this period. A lock down of recreational, sporting, and other youth activities that keep youngsters occupied may also be a contributing factor. During the Covid period, secondary school girls in rural western Kenya were more likely to be sexually active, less likely to report their first sexual encounter, and reported increased hours of non-school-related work (26). Higher rates of teenage pregnancies were also seen in SA in the past 2 years, possibly in relation to the Covid 19 pandemic (27).

Unprotected sex, either consensual or resulting from sexual co-ercion and exploitation, predisposes young girls to contracting sexually transmitted infections and HIV. Evidence shows that adolescent girls and young women have the highest rate of acquisition of HIV currently (28). Most teenage pregnancies are unplanned and often concealed, and not only have a disruptive effect on the schooling trajectory of children, but are also associated with complicated pregnancies, difficult labour, challenges with breastfeeding and issues with the ongoing healthcare of the baby.

### Mapping the way forward

Bold steps need to be taken and concerted efforts must be made to turn the tide of this trend. There should be a consolidation of efforts from various state and non-state actors to achieve a reduction in ABR's in SSA and globally. Data from South Asia, Middle East and North Africa indicates that ABR's dropped by between 75% and 80% by 2019 and continues to show a consistent decline (2). Activities that have proven to be effective in reducing adolescent pregnancy include the implementation of sexual and reproductive health policies, educational and vocational programs, empowerment initiatives, training activities, school retention programs and behaviour change campaigns (29).

Lessons can also be learnt from other countries which have had some success in reducing their ABR. In the Dominican Republic, a soft skills and vocational youth training program was shown to reduce the probability of teenage pregnancy by about 20% after implementation (30). In Mexico, the National Strategy for the Prevention of Adolescent Pregnancy (ENAPEA), a multisectoral approach is currently in the process of implementation to curb this phenomenon (31). In Northern Ireland live births from mothers aged 19 and younger dropped in 2020, largely due to progressive improvements in access to contraception and sexual education (32), and in the US the overall birth rate among 15- to 19-year-old girls dropped to half of what it had been in 2008 following various initiatives (33).

Similarly, many countries in SSA have developed and implemented national policies and programmes aimed at dealing with adolescent sexual and reproductive health (ASRH), including adolescent pregnancy (34).

In Ghana, the adolescent fertility rate declined steadily, and the country has also made progress in decreasing the rates of child marriage and school dropouts of girls (2). One of the key national policies for reducing adolescent pregnancy in Ghana is their Adolescent Health Service Policy and Strategy, which focuses on mainstreaming ASRH information, and gender-sensitive and responsive health services (35). These national policies coexist with programmes which are developed and implemented by both governmental and non-governmental organisations (36). In South Africa, the Department of Basic Education (DBE) has announced their implementation of comprehensive sexuality education (CSE) program in schools, which aims to empower young people with age-appropriate information about the cognitive, emotional, physical and social aspects of sexuality (37).

In Uganda, the Ministry of Education published revised guidelines for the prevention and management of teenage pregnancy in school settings, providing a policy to clarify the role of schools in adolescent pregnancies (38). At least 30 African Union countries now have laws, policies, and strategies in place that protect the rights of pregnant students and adolescent mothers to education (39).

Support grants are seen by some as a double edge sword in preventing teenage pregnancies. Perceptions that some recipients do not utilise the grant in the best interest of their children exist, and therefore need close monitoring by authorities. An alternate approach exists in Columbia, where a conditional cash transfer (CCT) program allows adolescent girls to receive a subsidy if they attended school, complete their school year, and enrol in the following year. This initiative was also effective in reducing pregnancy among adolescents across all grades included in the program (40).

A paradigm shift is required in the behaviour and attitude amongst teenagers themselves, supported by collective efforts from parents, teachers, healthcare providers and policy makers. Policymakers and community organisations should work in unison to develop and promote adolescent sexual and reproductive health policies and programmes, and emphasis should also be placed on human rights issues and gender empowerment programmes. The attitude of healthcare workers should be supportive rather than judgemental, and separate facilities should be considered for adolescent reproductive health services. Policy development, implementation strategies and quality assurance programmes are necessary to reduce adolescent pregnancies and meet the objectives of the United Nations Sustainable Development Goals (SDGs) (5).
